# Long-term association of ultra-short heart rate variability with cardiovascular events

**DOI:** 10.1038/s41598-023-45988-2

**Published:** 2023-11-03

**Authors:** Michele Orini, Stefan van Duijvenboden, William J. Young, Julia Ramírez, Aled R. Jones, Alun D. Hughes, Andrew Tinker, Patricia B. Munroe, Pier D. Lambiase

**Affiliations:** 1 Institute of Cardiovascular Science, University College London, 1-19 Torrington Pl, London, WC1E 7HB UK; 2 MRC Unit for Lifelong Health and Ageing at University College London, London, UK; 3 Barts Heart Centre, St Bartholomew’s Hospital, London, UK; 4 Clinical Pharmacology and Precision Medicine, William Harvey Research Institute, Faculty of Medicine and Dentistry, Queen Mary University of London, London, UK; 5 Big Data Institute, Nuffield Department of Population Health, University of Oxford, Oxford, UK; 6 Aragon Institute of Engineering Research, University of Zaragoza, Zaragoza, Spain; 7 Centro de Investigación Biomédica en Red, Bioingeniería, Biomateriales y Nanotecnología, Zaragoza, Spain; 8 NIHR Barts Biomedical Research Centre, Faculty of Medicine and Dentistry, Queen Mary University of London, London, EC1M 6BQ UK

**Keywords:** Arrhythmias, Cardiology, Biomedical engineering, Cardiovascular diseases

## Abstract

Heart rate variability (HRV) is a cardiac autonomic marker with predictive value in cardiac patients. Ultra-short HRV (usHRV) can be measured at scale using standard and wearable ECGs, but its association with cardiovascular events in the general population is undetermined. We aimed to validate usHRV measured using ≤ 15-s ECGs (using RMSSD, SDSD and PHF indices) and investigate its association with atrial fibrillation, major adverse cardiac events, stroke and mortality in individuals without cardiovascular disease. In the National Survey for Health and Development (n = 1337 participants), agreement between 15-s and 6-min HRV, assessed with correlation analysis and Bland–Altman plots, was very good for RMSSD and SDSD and good for PHF. In the UK Biobank (n = 51,628 participants, 64% male, median age 58), after a median follow-up of 11.5 (11.4–11.7) years, incidence of outcomes ranged between 1.7% and 4.3%. Non-linear Cox regression analysis showed that reduced usHRV from 15-, 10- and 5-s ECGs was associated with all outcomes. Individuals with low usHRV (< 20th percentile) had hazard ratios for outcomes between 1.16 and 1.29, *p* < 0.05, with respect to the reference group. In conclusion, usHRV from ≤ 15-s ECGs correlates with standard short-term HRV and predicts increased risk of cardiovascular events in a large population-representative cohort.

## Introduction

Heart rate variability (HRV)^1, 2^ is a cardiac autonomic marker^1, 2^, and low HRV is considered a marker of impaired autonomic function with established prognostic value in cardiac patients^3^. A limited number of population-based studies conducted in individuals without underlying cardiovascular disease^4^ have also demonstrated its association with cardiovascular events^5^, coronary heart disease^6^, atrial fibrillation^7, 8^ and all-cause mortality^6^. Some studies have suggested that both reduced and increased HRV may be associated with long-term risk of atrial fibrillation^7, 8^ and mortality^9^, but U-shaped associations require validation in larger cohorts.

According to current guidelines^2^, short-term HRV indices should be measured from ECG recordings lasting ≥ 5 min, which may limit its use at scale. To overcome this limitation, ultra short-term HRV (usHRV), i.e. the evaluation of HRV from ECG recordings of just 10–30 s, has been proposed, and several studies have suggested good agreement with standard short-term HRV^10–12^. The use of usHRV could have a significant impact on large population-based studies and on strategies for early risk stratification as standard 10–20 s ECGs are recorded in hundreds of millions of individuals each year worldwide^13^. Furthermore, wearable devices, such as smartwatches^14^, typically record ECGs for 10–30 s and could be used to measure usHRV. Although there are untapped opportunities to harness these innovations for better understanding and preventing cardiovascular disease at a population level, to the best of our knowledge, the association between usHRV and risk of future cardiovascular events has not been investigated.

The aim of this study was two-fold: First, we assessed the use of usHRV as a surrogate for standard HRV by evaluating the agreement between HRV measured from 6-min and 15-s ECGs in the National Survey for Health and Development (NSHD) study. Second, we evaluated the association of usHRV with long-term risk of atrial fibrillation, major adverse cardiac events (MACE), stroke, and mortality in a large sub-cohort of UK Biobank (UKB) participants without cardiovascular disease. We hypothesised that specific indices of usHRV measured from 15-s ECGs, namely the root mean square of successive differences (RMSSD), the standard deviation of successive differences (SDSD) and the high-frequency spectral power (PHF), could be used as surrogates for short-term HRV indices measured from ≥ 5-min ECGs, and that usHRV would be associated with multiple outcomes, reflecting the impact of autonomic dysfunction on a wide range of physiological processes.

## Results

**Figure 1:**
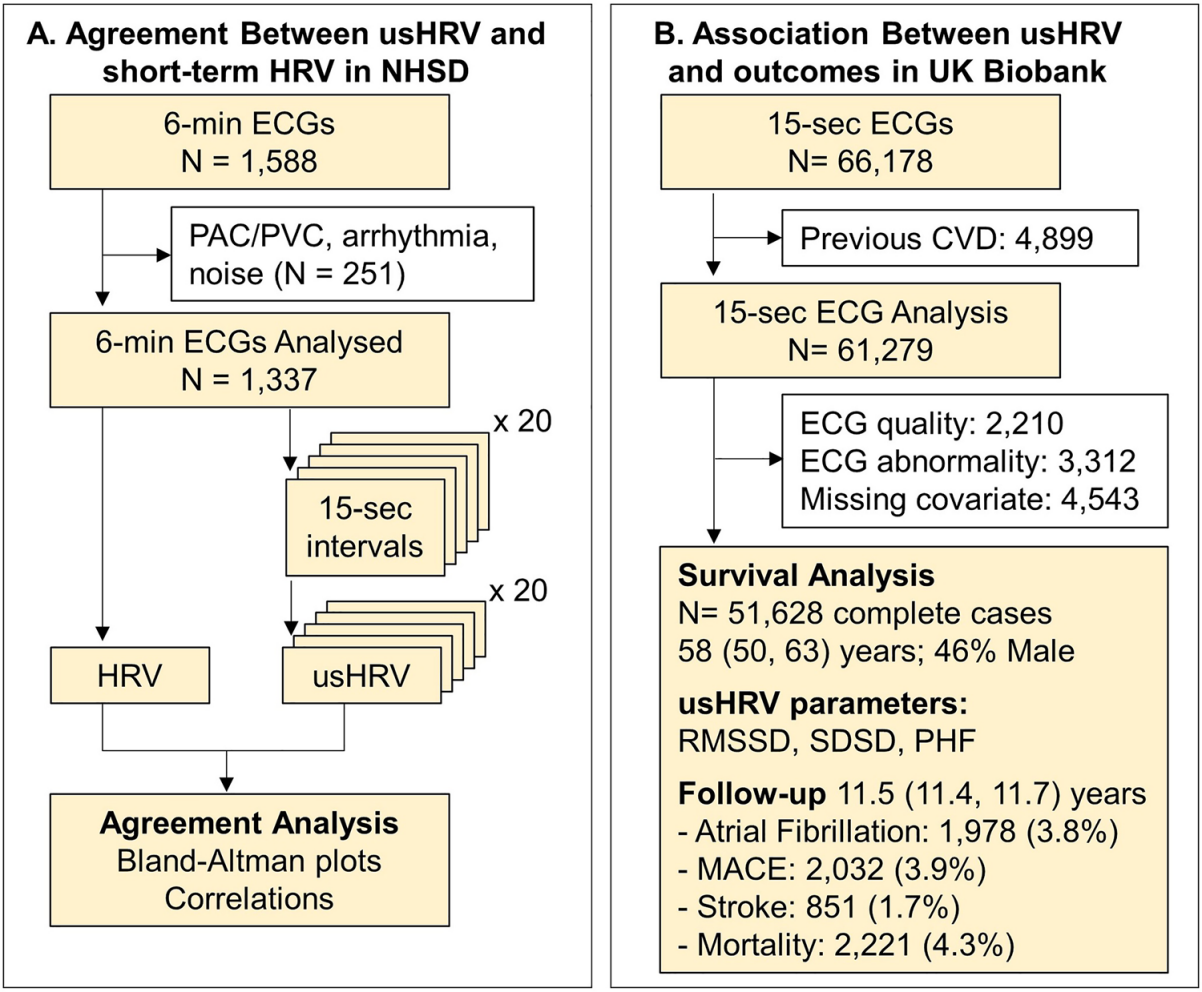
Flow diagram of the study. HRV: Heart rate variability; usHRV: Ultra-short HRV; PAC/PVC: Premature atrial/ventricular contractions; CVD: Cardiovascular disease. ECG abnormality includes bundle branch block morphology, sinus node disfunction, atrial fibrillation and premature atrial and ventricular contractions. MACE: Major adverse cardiac disease.

**Figure 2:**
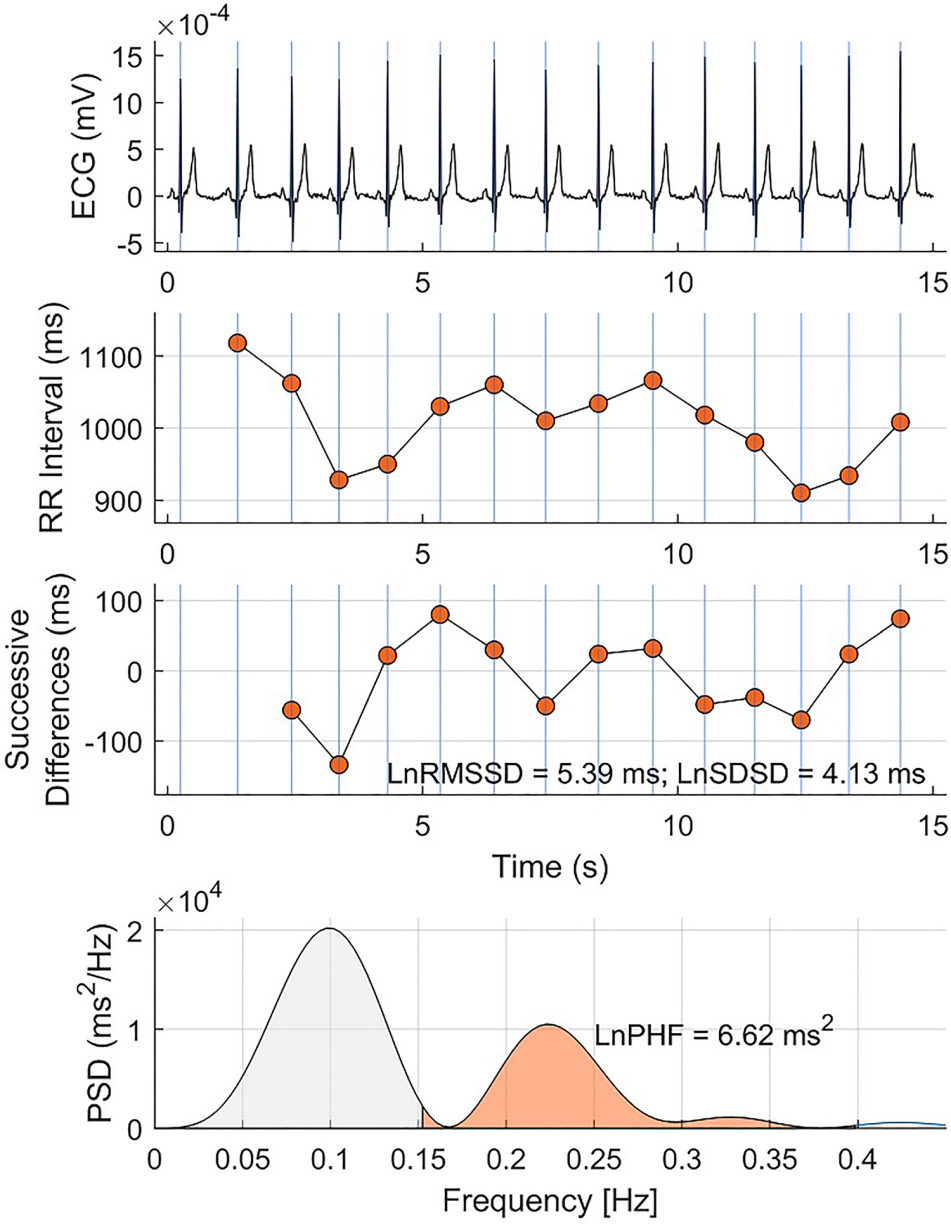
Heart rate variability indices. From top to bottom: a representative example of 15-s ECG, RR-interval (RRI), successive differences of RRI, power spectral density of RRI (after pre-processing, see text). The standard deviation of successive differences (SDSD), the root mean square of successive differences (RMSSD) and high-frequency power spectral density (PHF) for this example are shown in the figure.

The study design is represented in Fig. 1 and a representative example of a 15-s ECG recording and usHRV indices RMSSD, SDSD and PHF, is shown in Fig. 2. RMSSD, SDSD and PHF are well established HRV metrics^15^, which when measured over ≥ 5 min, are thought to reflect the overall autonomic modulation of heart rate (by RMSSD and SDSD), and respiratory sinus arrhythmia and parasympathetic activity (by PHF). RMSSD and SDSD were used because being based on successive differences, they measure fast heart rate oscillations that can be captured in ultra-short intervals. PHF was included because it captures heart rate oscillations with a period from 2.5 to 6.7 s (0.15–0.40 Hz), which theoretically can be measured satisfactorily from 15-s recordings.

### Agreement between standard short term and ultra-short HRV

**Figure 3:**
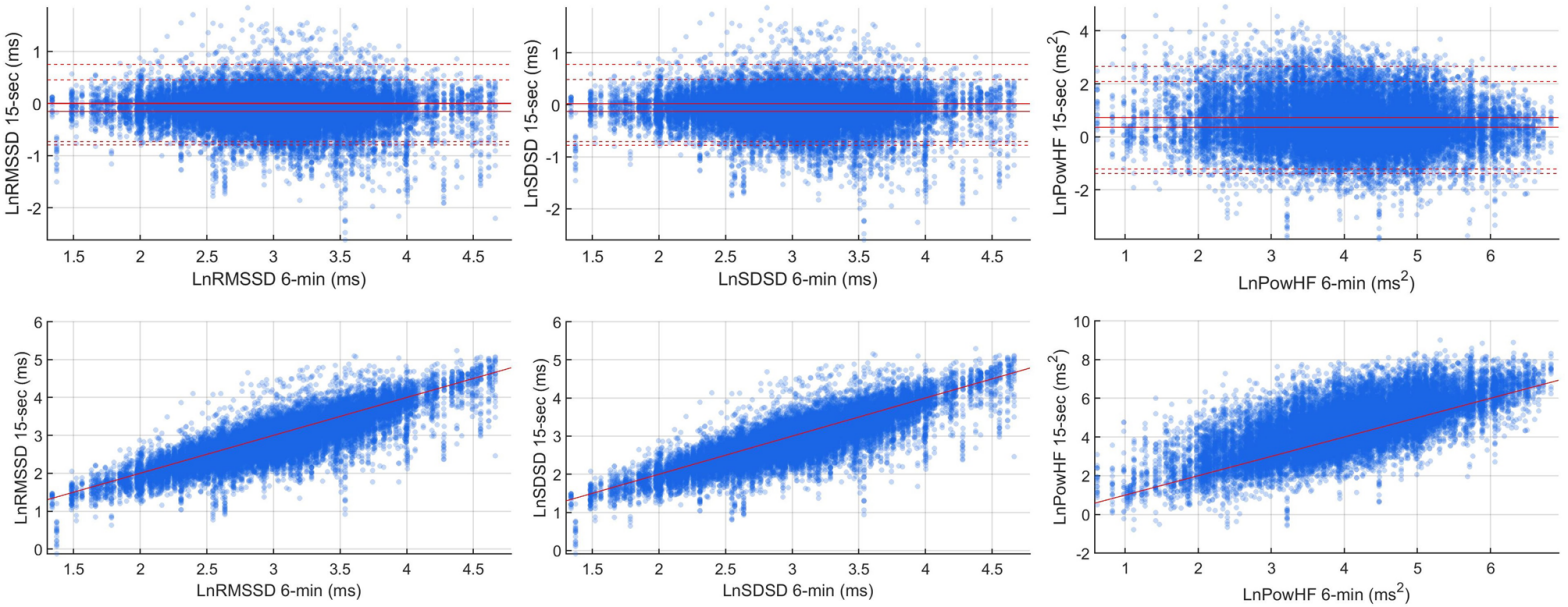
Method agreement. Bland–Altman (top) and scatter (top) plot comparing RMSSD and PHF from 6-min versus 15-s ECGs in NSHD. Data from 20 non-overlapping 15-s intervals are plotted against data from 6-min ECG recordings. Mean estimation error, limits of agreement and correlation coefficients were estimated for each non-overlapping 15-s intervals. In the Bland–Altman plot, solid red lines represent the minimum and maximum bias (mean estimation error) across the 20 15-s intervals, while dashed lines represent minimum and maximum limits of agreement across the 20 15-s intervals. In the correlation plots the red line represents the diagonal x = y.

Agreement between ultra-short (15 s) and standard short-term (6 min) HRV was assessed in n = 1337 individuals from NSHD (age 63.7 (63.0, 64.3) years, 54.1% females, Fig. 1A) using Bland–Altman plots and correlation coefficients. HRV from 6-min ECGs was compared with usHRV from 20 non-overlapping ECG segments from the same recording. The Spearman’s correlation coefficient (cc) was cc = 0.84 [0.83, 0.85] (median [interquartile range]) for both for RMSSD and SDSD, and cc = 0.70 [0.69, 0.71] for PHF (Fig. 3). Bland–Altman plots for RMSSD and SDSD showed virtually no bias (− 0.12 [− 0.13, 0.11] ms and − 0.10 [− 0.11, − 0.09] ms), narrow limits of agreement (median from − 0.76 to 0.52 ms for RMSSD, and from − 0.74 to 0.55 ms for SDSD) and no interaction between reference measures and estimation error (Fig. 3). PHF showed a small positive bias (0.46 [0.46, 0.49] ms^2^) and slightly larger limits of agreement (median from − 1.33 to 2.26 ms^2^). Frequency histograms of 15-s and 6-min HRV were similar (Supplementary Fig. S1). Differences in odds ratios for prevalent cardiovascular disease and diabetes when using standard versus usHRV were small, with mean absolute percentage error equal to 2.6% for RMSSD and SDSD, and 4.8% for PHF (Supplementary Fig. S2).

### usHRV cross-sectional associations with risk factors

usHRV was measured in 51,628 UKB participants without cardiovascular disease and showing normal sinus rhythm (54% women, aged 58 [50, 63], Fig. 1B). usHRV indices RMSSD, SDSD and PHF showed, after log-transformation, a Normal Gaussian distribution (Supplementary Fig. S3, Lilliefors test). Spearman’s correlation coefficient between RMSSD, SDSD and PHF ranged between 0.87 and 1.00, while correlation between usHRV indices and resting heart rate or heart rate recovery ranged between 0.41 and 0.51 (Supplementary Fig. S4).

**Figure 4:**
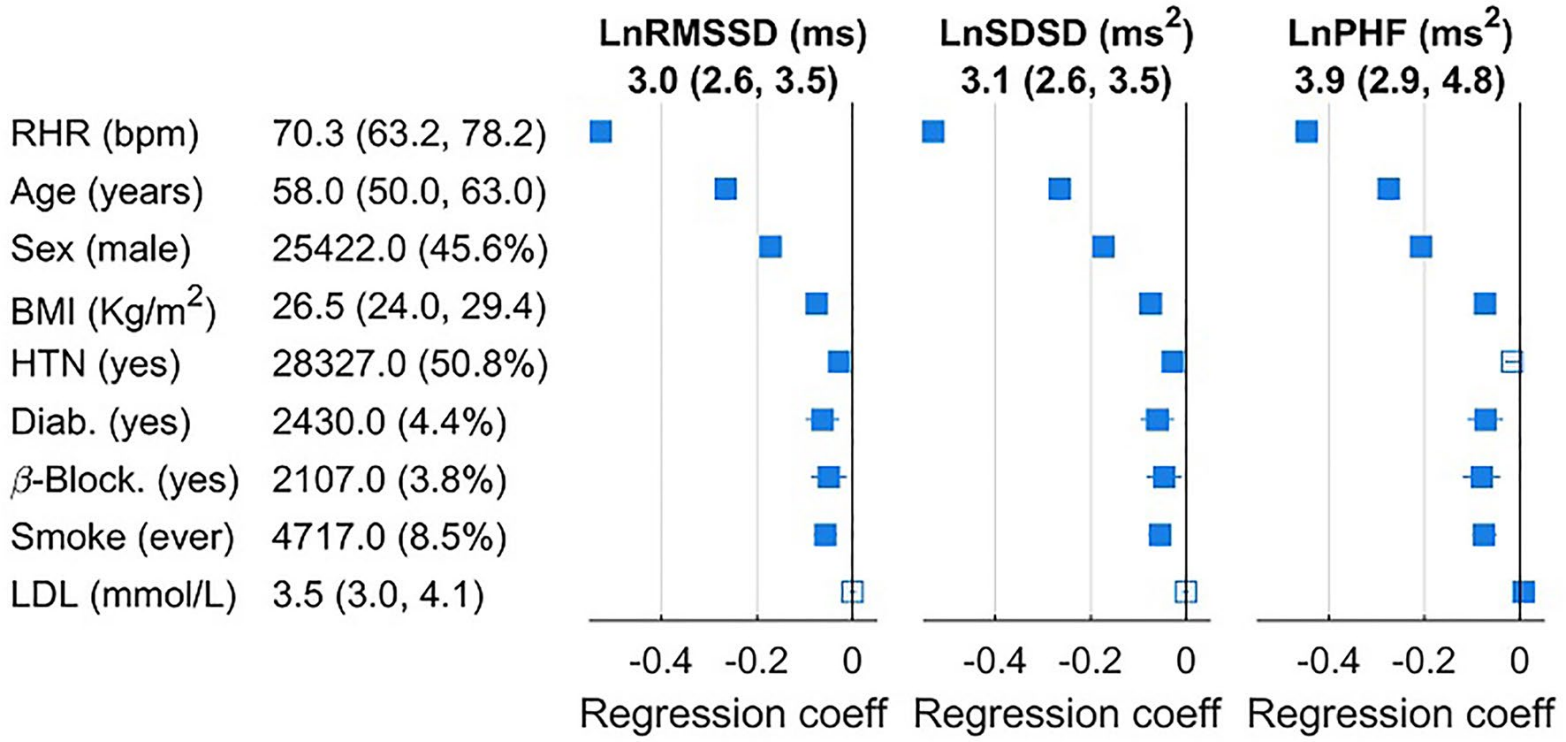
Cross-sectional associations between risk factors and usHRV. Distributions are shown as median (interquartile range). Forest plots show the estimated coefficients and confidence intervals of a linear multivariable regression models. Continuous variables, including usHRV indices, were normalised to mean = 0 and standard deviation = 1. BMI: Body mass index; HTN: Hypertension; β-Block: Use of beta-blockers; Diab: Type 2 diabetes. LDL: low-density lipoprotein cholesterol; RHR; resting heart rate.

Cross-sectional association between usHRV indices and main risk factors is reported in Fig. 4. As expected in view of its recognised interaction^16^, usHRV decreased with increasing resting heart rate. Age, body mass index, diabetes, use of beta-blockers, being male and smoking were also independently associated with lower usHRV.

### Association with prospective outcomes

**Figure 5:**
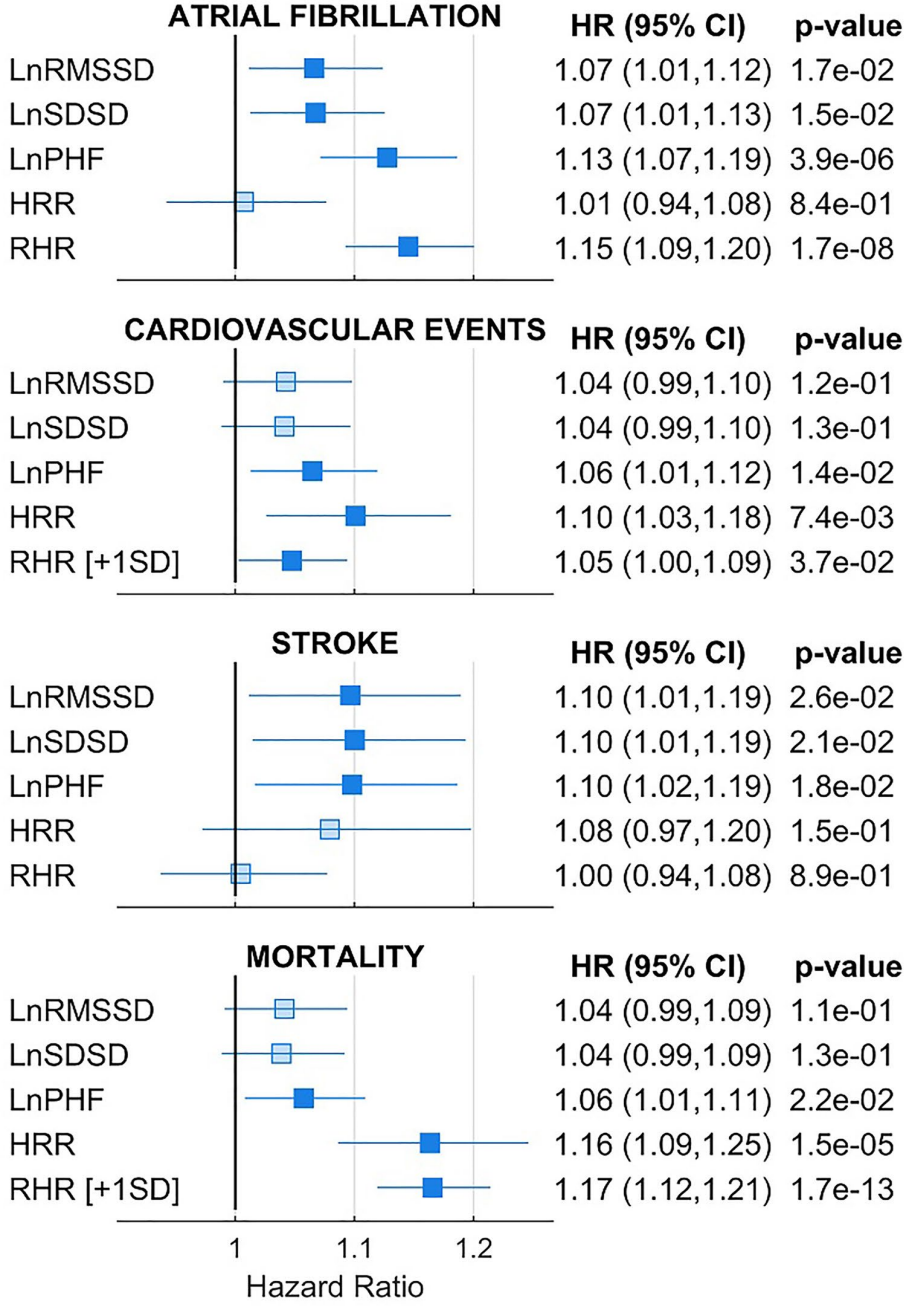
Long-term associations between usHRV and cardiovascular outcomes. Bars and whiskers indicate adjusted hazard ration and 95% confidence interval for 1 SD decrease in the continuous exposure unless specifically stated (+ 1SD indicates 1 SD increase). Models for ultra-short HRV indices (RMSSD, SDSD and PHF) and model for heart rate recovery (HRR) were adjusted for resting heart rate, age, sex, body mass index, hypertension, smoking, LDL cholesterol, diabetes, and use of beta-blockers.

After 11.5 (11.4–11.7) years, the incidences of atrial fibrillation, MACE, stroke, and mortality in UK Biobank participants were 3.8%, 3.9%, 1.7% and 4.3%, respectively. The predictive value of usHRV was assessed using linear and non-linear Cox regressions, which were adjusted for resting heart rate (RHR) and traditional risk factors including age, sex, body mass index, hypertension, smoking, LDL cholesterol, diabetes, and use of beta-blockers. For comparison, the predictive value of resting RHR and heart rate recovery from exercise was also measured. All usHRV markers were significantly associated with outcomes in linear unadjusted Cox regression models, with hazard ratios ranging between 1.25 and 1.40 per standard deviation decrease (Supplementary Fig. S5). In adjusted Cox linear models, at least one usHRV parameter remained inversely associated with all outcomes (Fig. 5). In particular, the risk for atrial fibrillation and stroke linearly increased with a decrease in all usHRV indices, with PHF showing the strongest association with atrial fibrillation (hazard ratio [95% confidence interval] = 1.13 [1.07–1.19], *p* < 0.01 per standard deviation decrease), and RMSSD, SDSD and PHF showing a similar association with stroke (1.10 [1.02–1.19] per standard deviation decrease, *p* < 0.02).

A standard deviation decrease in heart rate recovery was associated with increased risk of MACE (1.10, 1.03–1.18, *p* < 0.01) and mortality (1.16, 1.09–1.25, *p* < 0.01), but no association was found between heart rate recovery and atrial fibrillation or stroke.

**Figure 6:**
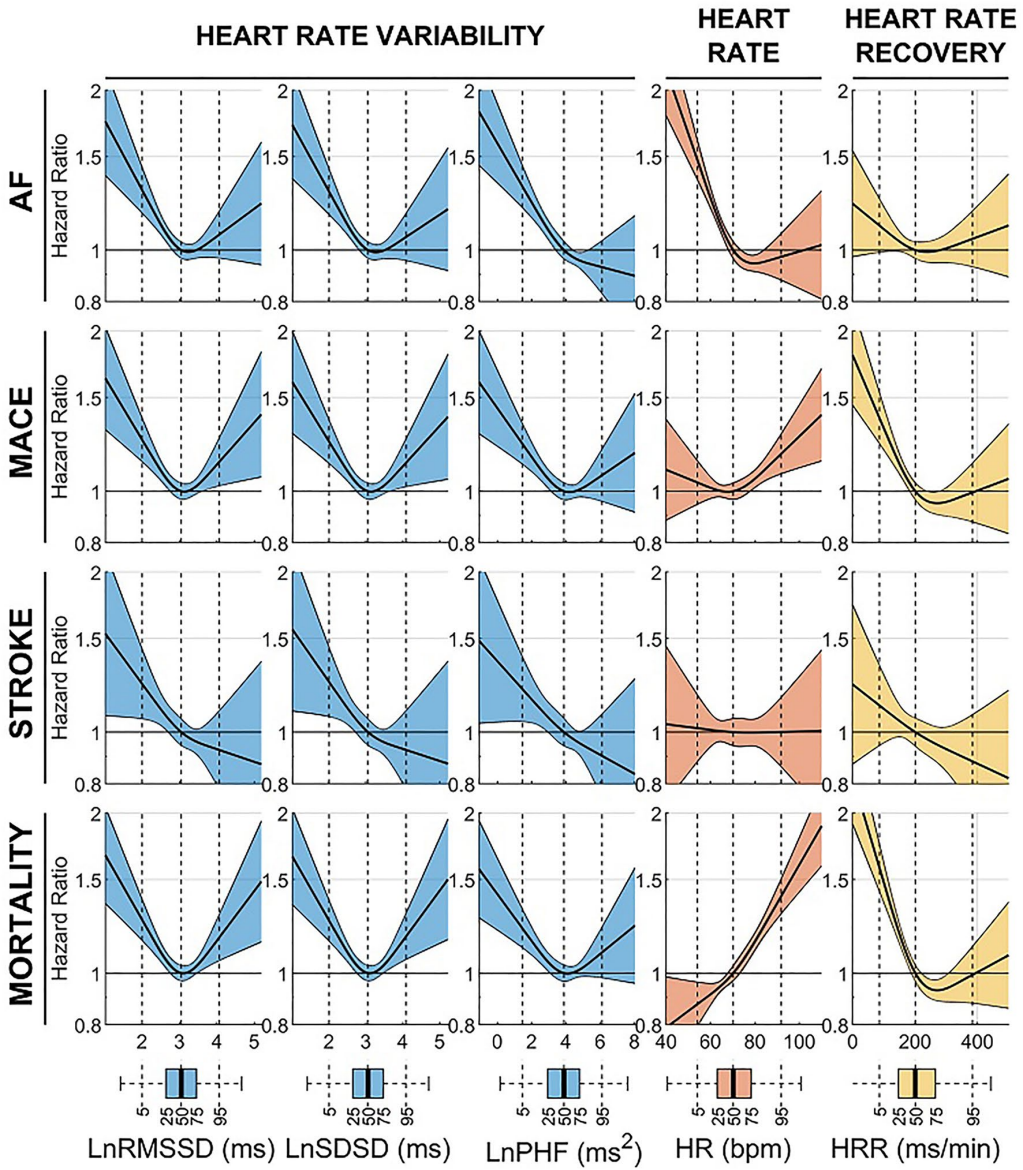
Non-linear relationships for long-term associations between usHRV and cardiovascular outcomes: Hazard ratio and 95% confidence interval (shaded area) for atrial fibrillation (AF), major adverse cardiac events (MACE), stroke and mortality are shown as a function of ultra-short heart rate variability (usHRV, RMSSD, SDSD, PHF), resting heart rate, and heart rate recovery (HRR). Boxplots represent exposures’ distribution, with number indicating percentiles. All models were adjusted for standard risk factors (see text) and usHRV and HRR models were further adjusted for resting heart rate.

Since U-shaped associations between HRV and outcomes have been previously reported^7–9^, the risk associated with low or high usHRV was further assessed using non-linear Cox regression models (Fig. 6). A significant association between reduced usHRV and increased risk for atrial fibrillation, MACE, stroke, and mortality was confirmed (confidence intervals of hazard ratios > 1 for low usHRV values) for all usHRV indices. A U-shaped association was found between RMSSD and SDSD, and MACE and mortality, which may explain why the same indices did not show a significant association with MACE and mortality using linear Cox regressions (for which the hazard ratio showed confidence intervals between 0.99 and 1.10, Fig. 5). U-shaped trends as well as the significant association between reduced usHRV and MACE and mortality were confirmed by comparing usHRV across quintiles. Compared to individuals with RMSSD within the 2nd and 4th quintiles (reference group), those with RMSSD in the lowest quintile showed an adjusted hazard ratio of 1.29 [1.10–1.51], *p* < 0.01, for MACE, and 1.16 [1.01–1.34], *p* = 0.04, for mortality (Supplementary Table S3, Supplementary Fig. S6).

The analysis of associations with conditions underlying MACE, showed a similar trend for heart failure, myocardial infarction, and life-threatening ventricular arrhythmia (Supplementary Fig. S7).

Sensitivity analysis showed a similar non-linear association between usHRV and outcomes after excluding participants with diabetes or using beta-blockers (Supplementary Fig. S8).

### Impact of recording duration

**Figure 7:**
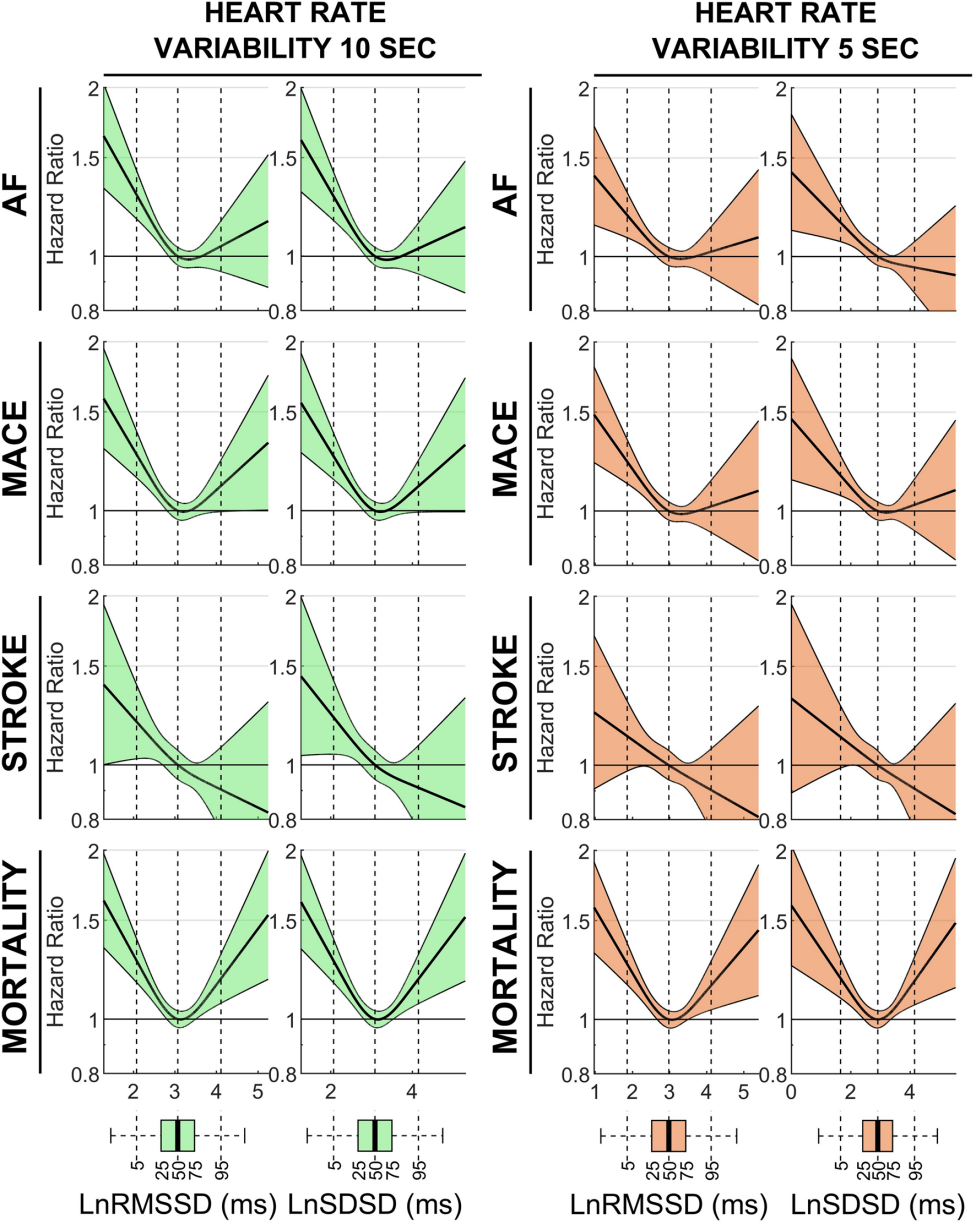
Associations between 10-s and 5-s HRV and outcomes. Hazard ratio and 95% confidence interval (shaded area) for atrial fibrillation (AF), major adverse cardiac events (MACE), stroke and mortality are shown as a function of ultra-short heart rate variability. Boxplots represent exposures’ distribution, with number indicating percentiles. All models were adjusted for standard risk factors (see text). The root mean square of successive differences (RMSSD) and the standard deviation of successive differences (SDSD) were measured during the first 10 s (left, green) and 5 s (right, orange) of the resting phase.

RMSSD and SDSD, but not PHF, could be measured from recordings lasting less than 15-s. The association between ultra-short HRV parameters measured from the first 10-s or 5-s of the recordings and outcomes was similar, but slightly attenuated, compared to the association between 15-second HRV and outcomes (Fig. 7). This is likely because RMSSD and SDSD measured from 10- and 5-s recordings correlated well with indices measured from 15-s recordings (0.95 and 0.82 for RMSSD, and 0.95 and 0.72 for SDSD, respectively).

## Discussion

The aim of this study was to provide a comprehensive assessment of long-term associations between cardiac autonomic function and multiple health outcomes in individuals without cardiovascular disease using HRV indices measured in ≤ 15-s ECGs (usHRV). The main findings are: (1) Agreement between ultra-short (15-s) and short-term (6-min) HRV indices was very good for RMSSD and SDSD, and moderate for PHF. (2) Low 15-s HRV was associated with increased long-term risk of atrial fibrillation, major adverse cardiac events, stroke, and mortality, independently of resting heart rate and standard cardiovascular risk factors. In adjusted models, reduced usHRV carried a similar long-term risk for atrial fibrillation, MACE, and mortality as standard risk factors such as diabetes, hypertension, and smoking. (3) RMSSD and SDSD derived from 10- or 5-s recordings showed similar associations with outcomes.

These data provide support for usHRV as a preclinical marker for multiple clinical outcomes. The prognostic value of reduced HRV (measured from recordings much longer than 15-s) was previously demonstrated in population-based cohorts including ARIC^6, 7, 17^, Framingham^5, 18^ , MESA^8^ and confirmed in a meta-analysis of eight studies^4^. Previous studies have also demonstrated a good agreement between standard and ultra-short HRV, especially for RMSSD^10–12^. This is the first study to simultaneously validate ultra-short HRV metrics and to demonstrate its predictive value for multiple outcomes in a very large population-representative study. Most of previous studies focused on a single outcome and derived HRV from longer ECG recordings (24 h^19^, 2 h^5, 18^ and 2 min^6, 7^) with only two studies using 10–15 s ECGs^8, 9^, but in smaller cohorts.

### Agreement between ultra-short and short-term HRV

Our analysis of 1337 NSHD participants showed that the agreement between 6-min and 15-s HRV indices was very good for RMSSD and SDSD and good for PHF. We also demonstrated that the use of 15-s instead of 6-min HRV did not substantially impact on regression models assessing association with prevalent cardiovascular disease. A very good agreement for RMSSD and SDSD is expected because these are based on successive differences of normal heartbeats and capture fast changes in the heart rate, which are related to parasympathetic tone^15^, while PHF, which assesses respiratory-related oscillations with period ranging between 2.5 and 6.7 s, may be affected by reduced spectral resolution when resting heart rate is particularly low. Of note, RMSSD and SDSD measured from 10-s ECGs were very similar (correlation coefficient = 0.95) with those measured from 15-s ECGs. In this study, ultra-short HRV measures have been accepted as cardiac autonomic markers because of their correlation with standard short-term HRV indices. Further studies are needed to fully determine the relationship between autonomic activity and resting ultra-short HRV.

### Comparison with previous studies on HRV and incident outcomes

An association between HRV and incident atrial fibrillation was found in previous, smaller, studies^7, 8, 20^. Two studies reported U-shaped associations between HRV and incident atrial fibrillation^7, 8^, which were not confirmed by our data. In Supplementary Fig. S9, we demonstrate that an apparent association between high usHRV and atrial fibrillation can be produced when just few individuals (accounting for 0.2–0.6% of the study population) without known cardiovascular disease but with premature atrial contractions in the 15-s ECG were included in the analysis. This suggests that premature atrial contractions, which are strongly associated with incident atrial fibrillation^21^ and which dramatically, but artifactually, increase HRV if not removed, may explain previously reported association between high HRV and atrial fibrillation. Our data however showed a U-shaped association between RMSSD or SDSD, and MACE and mortality even after rigorously including only individuals in normal sinus rhythm. Similar findings were reported in the Rotterdam study^9^ and further investigation is required to clarify this association. Data on HRV and incident stroke is also limited, with only few studies reporting associations in population based cohorts^22, 23^. Our findings should encourage further investigation on autonomic dysfunction and risk of stroke in individuals without known cardiovascular disease.

The fact that significant associations were found for different health outcomes can be explained by the role that the autonomic nervous system plays in multiple aspects of cardiac and cardiovascular function. The mechanisms whereby autonomic dysfunction increases the risk of atrial fibrillation, MACE, stroke, and mortality are not completely understood and require further investigation.

### Potential applications and clinical impact

The use of HRV indices derived from ultra-short recordings may open new opportunities for autonomic nervous system assessment at scale, because it can be measured from standard clinical ECGs, which are taken in millions of individuals every day. This number is expected to grow dramatically thanks to popular wearable devices, including smartwatches and mobile Apps^24^, which usually record the ECG for just few seconds. Interestingly, in this study, usHRV showed a similar association with MACE and mortality as heart rate recovery, an established cardiac autonomic marker and risk-predictor which however requires a standardised exercise stress test. Although our findings may have a limited impact on patient-specific clinical care, ultra-short HRV can improve our understanding of cardiovascular disease mechanisms at the population level, including providing biological insight into the interaction and causal links between cardiac autonomic dysfunction and cardiovascular disease^25^. In our study, the ECG was recorded while participants sat on a bicycle for 15 s. HRV in a sitting position is expected to be lower than in the recommended supine position^2^, and we cannot exclude that the association between HRV and cardiovascular outcomes is affected by posture and by specific experimental settings.

#### Strengths, limitations, and future directions

This study has several strengths. It included both cross sectional analyses to validate usHRV indices and longitudinal analysis of a large prospective cohort (UKB, n = 51,628) for investigating interaction with outcomes. The follow-up period was long (over 11 years), and we used linear and non-linear regression models adjusted for multiple risk factors to study associations with multiple outcomes. ECG data analysis used state of the art signal processing, and visual inspection of 34,561 ECGs (61% of total) by expert reviewers in a process designed to accurately identify abnormal ECGs (estimated 0% false positive rate and 0.06% false negative rate)^21^. Several limitations need to be acknowledged. Several HRV indices^1^, e.g. SDNN or spectral indices associated with slower heart rate oscillations, cannot be measured from 15-s ECGs, and this may reduce the predictive value of ultra-short HRV compared to standard > 5-min HRV. Hospital episode statistics may underestimate the true incidence of outcomes and some delay may exist between an event and the date of its reporting. There is evidence of “heathy volunteer” selection bias in the UK Biobank, which may not be representative of the general population. Due to the limited number of events, myocardial infarction, heart failure and life-threatening ventricular arrhythmia were combined in the aggregate outcome MACE. However, a significant association between reduced uHRV and incident heart failure or myocardial infarction, the main components of MACE, was also found. Finally, in this study we investigated ultra-short HRV at rest. HRV is usually measured at rest as a marker of autonomic tone^2^. However, assessment of the autonomic nervous system response to stressors such as exercise^26–30^ or tilt table test^31^, which is characterised by a dynamic interplay of sympathetic and parasympathetic withdrawal and reactivation^28^, could provide additional insights into cardiovascular health and complementary prognostic information. For example, a recent study has shown that cardiovascular variability measured during an orthostatic challenge predicts mortality better than resting cardiovascular variability^31^. Future studies will need to investigate the predictive value of the ultra-short HRV response to an autonomic challenge.

## Conclusion

Reduced ultra-short HRV from ECGs recordings lasting between 5 and 15 s was associated with increased risk of atrial fibrillation, MACE, stroke and mortality in participants without cardiovascular disease. With standard ECGs being measured in hundreds of millions of patients every year worldwide, and wearable technology transforming the ECG into a ubiquitous test, usHRV could make an impact on our understanding of cardiovascular disease and its prevention at the population level and requires further investigation.

## Methods

### HRV parameters

UsHRV was measured using established metrics^2^ including the root mean square and standard deviation of successive differences (RMSSD and SDSD, respectively), reflecting the overall autonomic modulation of heart rate, and high-frequency spectral power (PHF), which reflects respiratory sinus arrhythmia and parasympathetic activity. To measure PHF, the time-series of RR-intervals was evenly interpolated at 4 Hz, high-pass filtered (cut-off 0.03 Hz) to derive its variability. Its power spectral density was estimated using the Fast Fourier transform and PHF was measured in the spectral band 0.15–0.40 Hz. All usHRV indices were log transformed to account for skewed distributions, as in previous studies^7, 8^. An example of a 15-s ECG recording and corresponding HRV indices from the UK Biobank (UKB) study is shown in Fig. 2.

ECGs were analysed using algorithms developed by our group^21, 32–34^. Since HRV indices are sensitive to abnormal heart rhythm, ECGs and RR-intervals were carefully examined to ensure that only recordings acquired in normal sinus rhythm were included. The procedure is detailed elsewhere^21^ and briefly described here. In UKB, all ECGs showing SDSD, RMSSD or PHF larger than the 70^th^ indices’ percentile or flagged by an algorithm designed to identify premature ventricular contractions (N = 19,561) were reviewed on a Graphical User Interface (Matlab 2022a, Mathwork). Initial visual revision was performed by one expert, with diagnoses of abnormal ECG (including bundle-branch block morphology, premature atrial or ventricular contractions, sinus node dysfunctions, atrial fibrillation, and noise) confirmed by a second expert. Ambiguous cases were discussed with a third expert to reach a consensus. ECGs with RMSSD, SDSD or PHF < 70th percentile and not automatically flagged as containing premature ventricular contractions (N = 37,175) were considered to show normal sinus rhythm. To assess how many of these ECGs could be abnormal (false negative rate), 15,000 of them were randomly selected and manually reviewed. Of these, 9 were abnormal, which corresponds to a false negative rate of 0.06%. This suggests that only 13 abnormal ECGs may have been erroneously accepted as normal among the remaining 22,175 ECGs not manually revised.

Resting heart rate and heart rate recovery were included in the analysis as comparison. Heart rate recovery was measured in milliseconds as the difference between the RR interval at 1 min recovery and RR interval at peak exercise, as in previous studies^32, 33^.

### Correlation between usHRV and standard HRV in NSHD study

The NSHD study recruited a representative sample of 5362 men and women born in England, Scotland and Wales in a single week in March 1946^35^. Ethical approval for the study investigations at 60–64 years was given by the Central Manchester Research Ethics Committee (07/H1008/245) and by the “Scottish A Research Ethics Committee” (08/MRE00/12). All methods were performed in accordance with the relevant guidelines and regulations. A 6-min resting 3-lead ECG (sampling frequency 600 Hz) was recorded in 1588 participants. After excluding participants showing more than 1 ectopic every 2 min, ECG recordings were available in N = 1337 participants (data reported in Supplementary Table S4). In these recordings, ectopic beats were removed and interpolated. Standard HRV parameters were derived from the entire recordings, while usHRV parameters were measured in 20 non-overlapping intervals of 15-s duration from the same recordings. Comparison between 6-min and 15-s HRV indices was conducted with Bland–Altman plots and by measuring the Spearman’s correlation coefficient. The impact of using 15-s versus 6-min HRV in statistical modelling was assessed by comparing odds ratios for prevalent cardiovascular disease and diabetes derived from logistic regressions adjusted for age, sex, and body mass index.

### Prognostic value of usHRV in the UK Biobank

The UK Biobank study has approval from the North West Multi-Centre Research Ethics Committee, and all participants provided informed consent^36^. Ethical approval for this study was granted through UK Biobank application 8256.

Participants from the UK Biobank who underwent an ECG test during 2009–2010 and 2012–2013 were included in this study.

The ECG was recorded during the 15-s resting phase preceding a standard ECG exercise stress test (protocol details are available on-line^37^). The participants were asked to sit on a stationary bike with the seat height adjusted to their comfort and their feet in the foot straps on the pedals. The ECG (Lead I) was recorded with a GE CardioSoft device at a sampling frequency of 500 Hz.

Figure 1 provides an overview of the study. ECG recordings were available in N = 66,178 participants. We excluded N = 4899 participants with prevalent cardiovascular disease defined from a hospital episode statistics code (Supplemental Table S1), self-report and inability to exercise. We also excluded participants not showing normal sinus rhythm as described in the previous section, including if the ECG signal quality was insufficient to discriminate between sinus and abnormal rhythm. Finally, we excluded participants with missing covariates. In total, 51,628 (54% women, aged 58 [50, 63]) individuals without cardiovascular disease were considered for complete case analysis.

Outcome data were derived from the linked hospital in-patient and mortality data through NHS services and national death registries^38^ coded in ICD-10 format (Supplementary Table S2)^39, 40^. Endpoints were (1) atrial fibrillation and atrial flutter; (2) major adverse cardiac events (MACE), a composite endpoint aggregating myocardial infarction, heart failure and life-threatening ventricular arrhythmia (results for single outputs are provided as supplementary material); (3) Stroke and transient ischemic attacks (reported as stroke in the following); and (4) Mortality from any cause.

Survival analysis was conducted using Cox regression and restricted cubic splines were used to model possible non-linear associations between usHRV and incident health outcomes^7, 8^. Models were adjusted for resting heart rate (RHR) and traditional risk factors including age, sex, body mass index, hypertension, smoking, LDL cholesterol, diabetes, and use of beta-blockers. In sensitivity analysis, data were analysed after excluding participants taking beta-blockers or with diabetes at the time of testing (N = 3933).

### Supplementary Information

Supplementary Information 1.

Supplementary Information 2.

## Data Availability

UK Biobank data will be returned to the UK Biobank that will make them available to researchers (https://www.ukbiobank.ac.uk/). Deidentified data and documentation on NSHD are available from https://www.nshd.mrc.ac.uk/data.
