# Supplementary material for: Long-term association of ultra-short heart rate variability with cardiovascular events
Source: Sci Rep. 2023 Nov 3;13:18966. doi: 10.1038/s41598-023-45988-2 (PMC10624663; doi:10.1038/s41598-023-45988-2)
Supplement: Supplementary file 1 — Supplementary Information 1. [file 41598_2023_45988_MOESM1_ESM.pdf]

Long-term association of ultra-short heart rate variability with cardiovascular events

Michele Orini *et al*

Supplementary Figure 1 2
Supplementary Figure 2 3
Supplementary Figure 3 4
Supplementary Figure 4 5
Supplementary Figure 5 6
Supplementary Figure 6 7
Supplementary Figure 7 8
Supplementary Figure 8 9
Supplementary Figure 9 10

Supplementary Figure 1

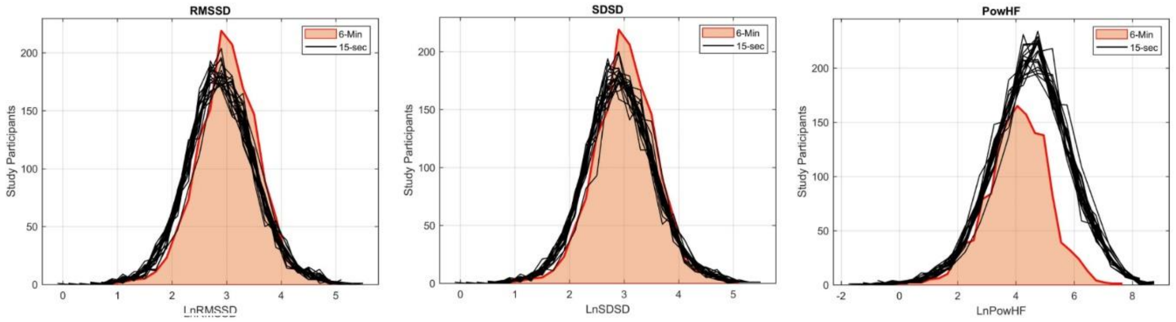

Supplementary Figure 1: Comparison of histograms showing the log-transformed distribution of 6-minute (orange shade) and 15-second (20 continuous black lines) HRV indices in NSHD. From left to right: RMSSD, SDSD and PHF.

Supplementary Figure 2

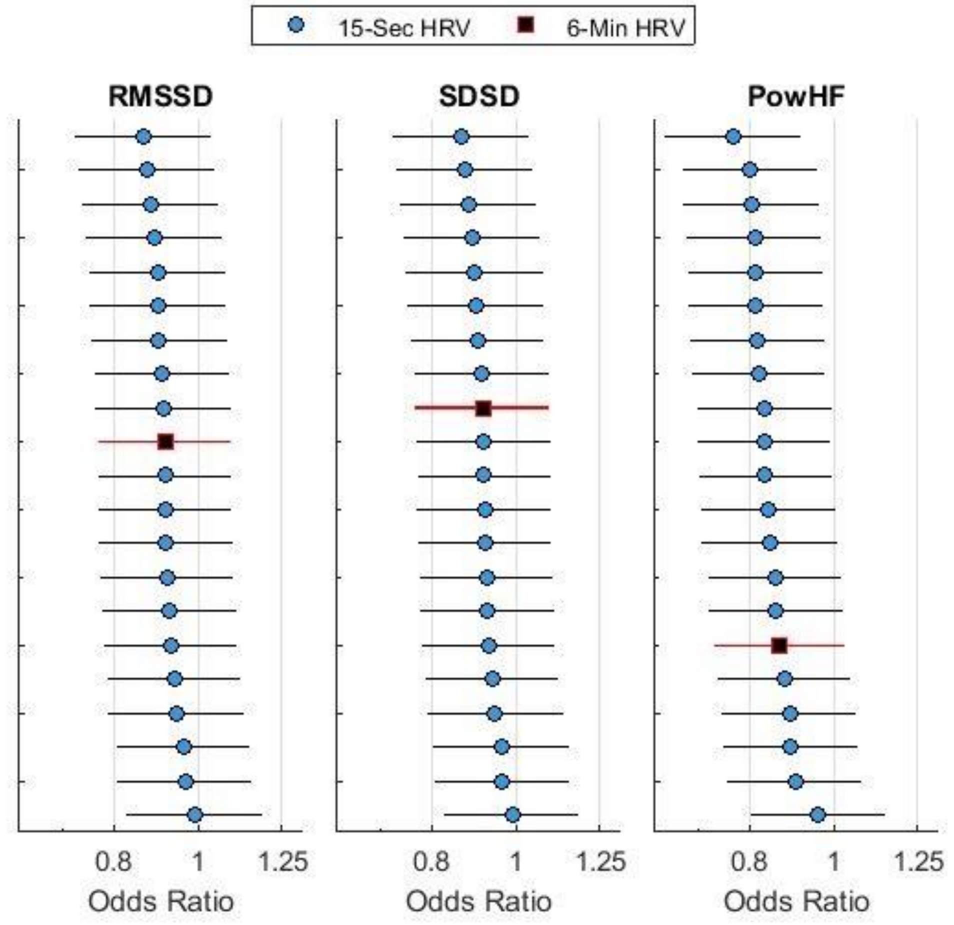

Supplementary Figure 2: Odds ratio (markers) and 95% confidence intervals (lines) for prevalent cardiovascular disease and diabetes (aggregate outcome with 9.8% prevalence) in NSHD. Logistic regressions were adjusted for sex, age, and body mass index. Odds ratio obtained using 6-minute HRV are reported as black squares, and results obtained using 15-second HRV are reported as blue circles (N=20 non-overlapping segments). The mean absolute percentage error when using 15-second HRV instead of 6-minute HRV is reported in the text. Data are reported ranked from lowest to largest odds ratio.

Supplementary Figure 3

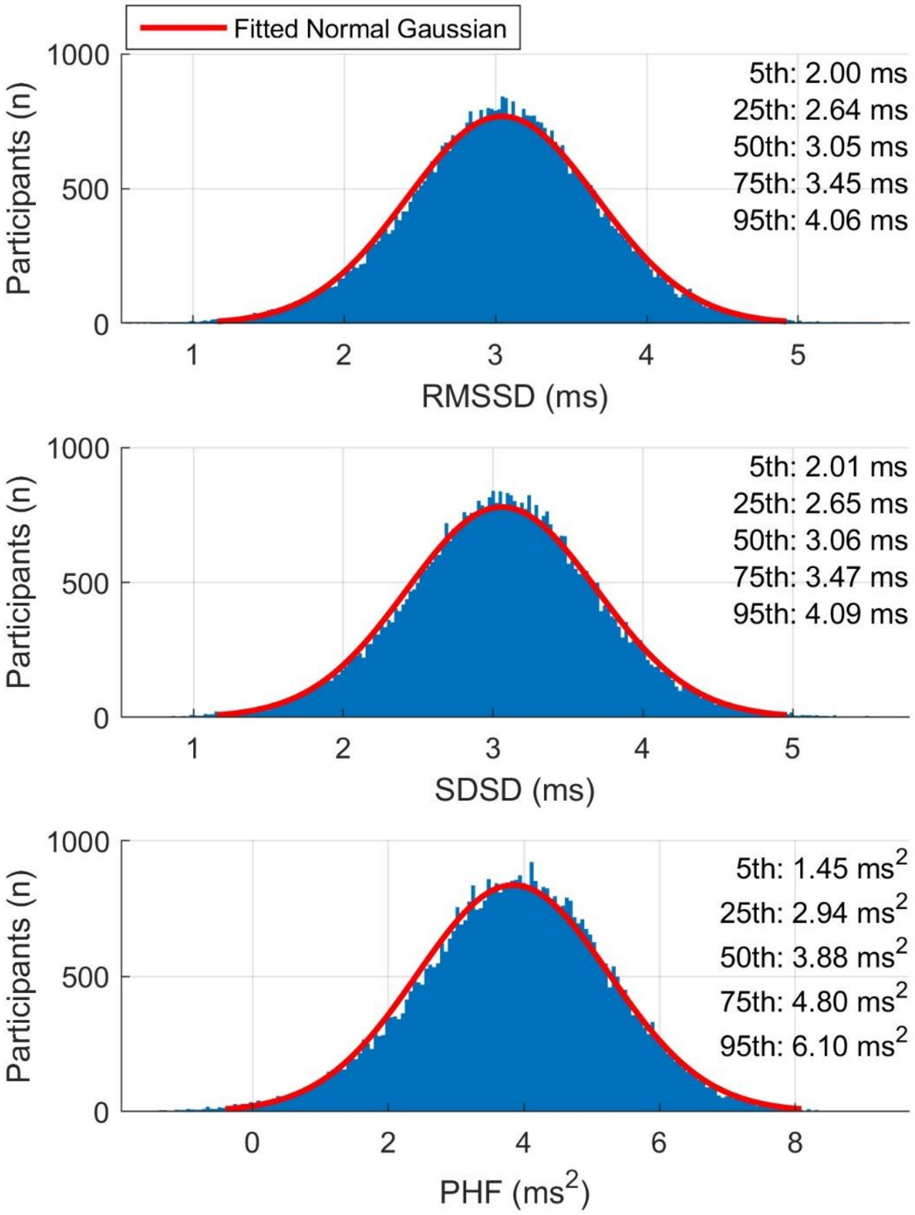

Supplementary Figure 3: Histograms showing log-transformed distribution of ultra-short HRV indices RMSSD, SDSD and PHF in UK Biobank (N=51,628). The red line represents a Normal Gaussian distribution fitted on the data. Median (50th percentile), interquartile range (25th – 75th percentile) and 5th – 95th percentile range are shown in the right-hand side.

Supplementary Figure 4

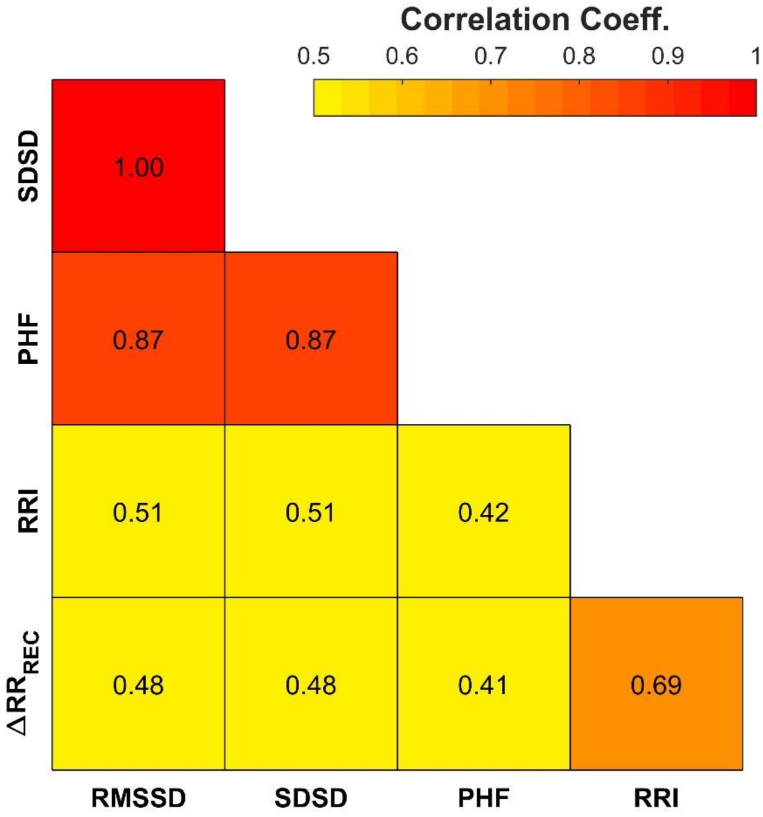

Supplementary Figure 4: Spearman's correlation coefficients (left) for usHRV parameters in UK Biobank (N=51,628 individuals). RMSSD: Root mean square of successive differences. SDSD: Standard deviation of successive differences. PHF: High power component of RR intervals. RRI : Resting RR intervals (median of RRI in 15 second recordings); ΔRR_{REC} : Heart rate recovery measured as differences of RRI after 1 min recovery and RRI at peak exercise.

Supplementary Figure 5

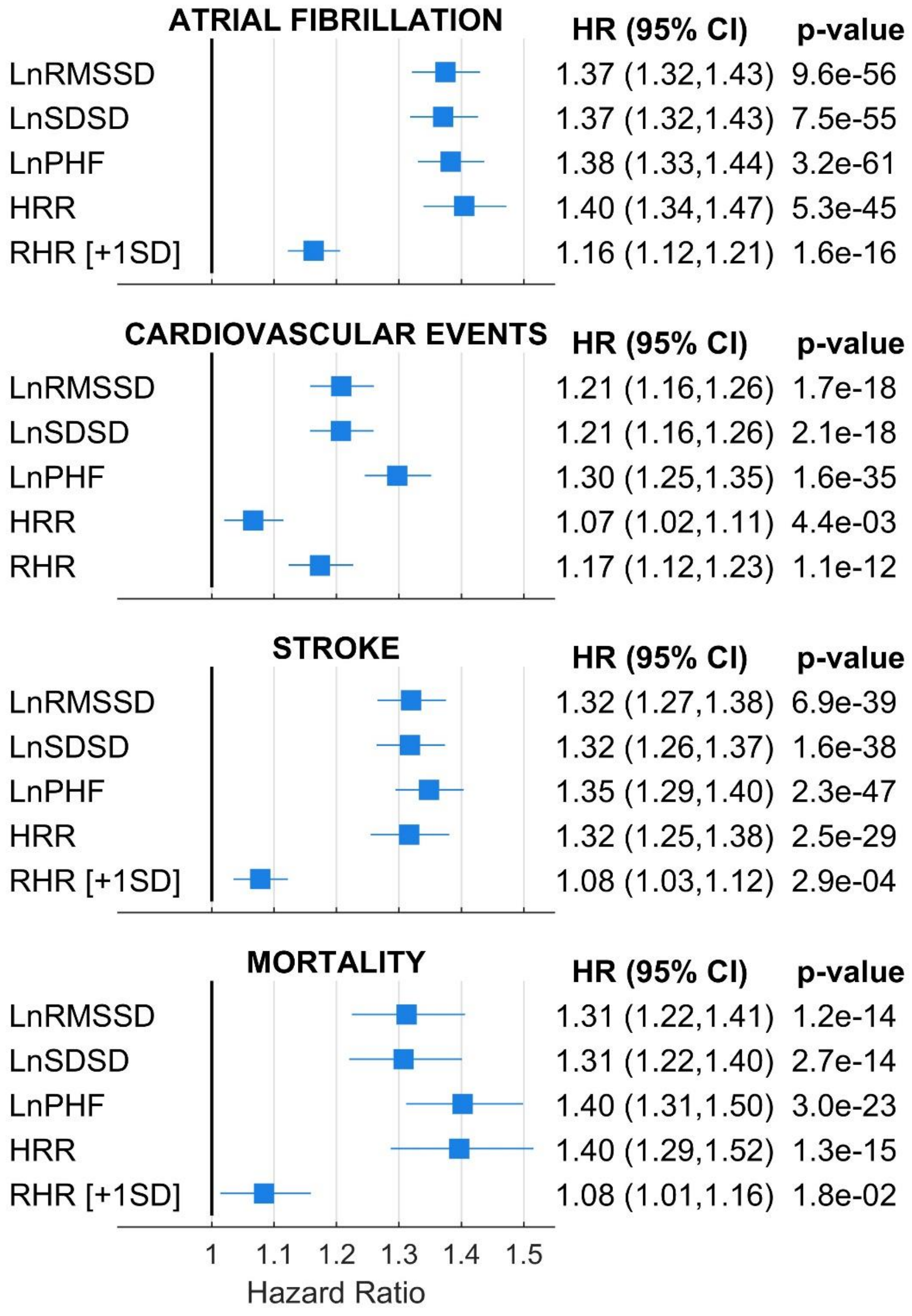

Supplementary Figure 5: Unadjusted models. Hazard ratios (95% confidence intervals) are shown for 1 standard deviation decrease in the exposure, unless specified as +1SD (1 standard deviation increase in the exposure).

Supplementary Figure 6

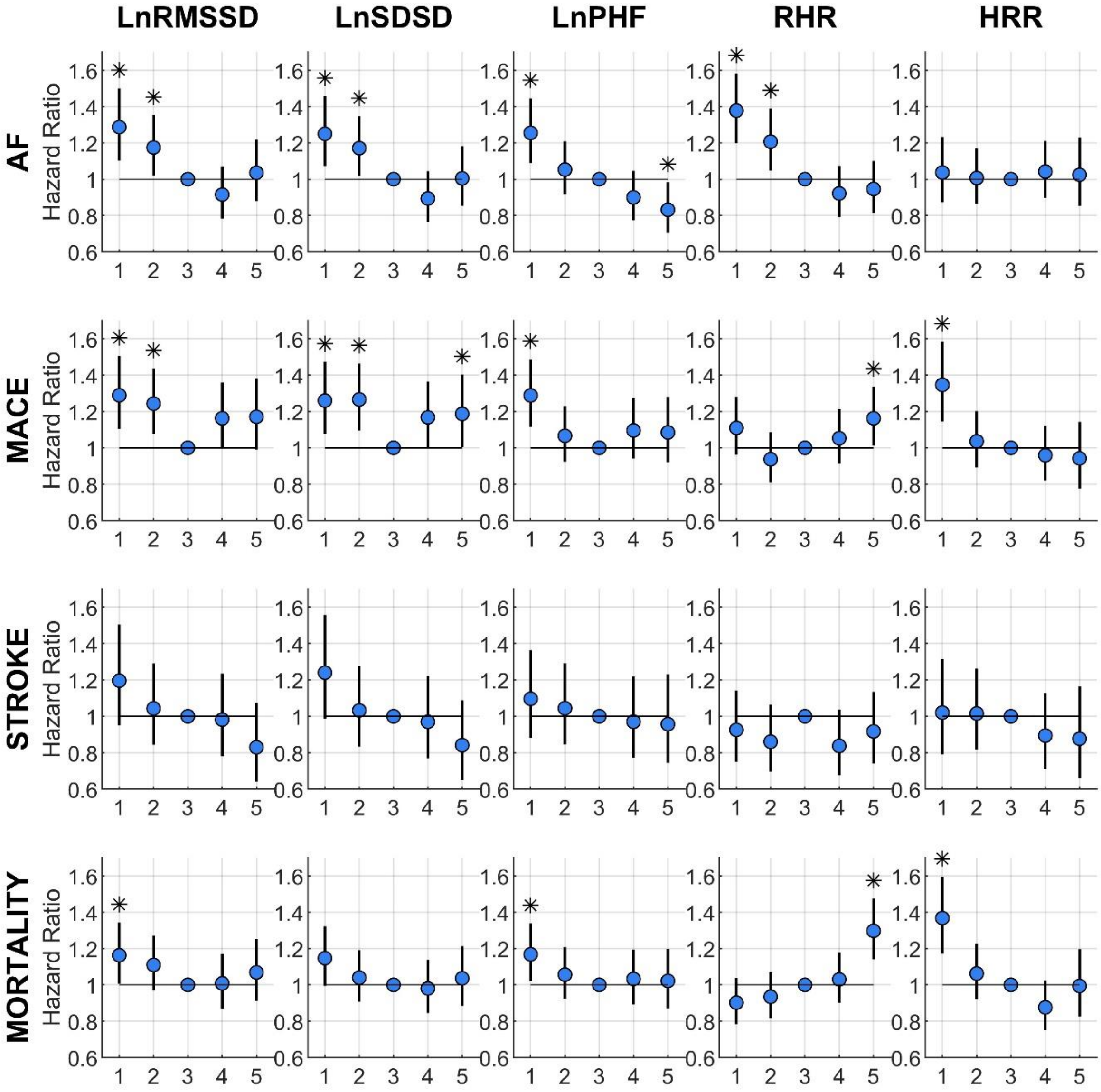

Supplementary Figure 6: Adjusted Cox regression models stratified per usHRV quintiles. Hazard ratios (95% confidence intervals) for each quintile are computed using the 3rd quintile as reference. *: $p<0.05$.

Supplementary Figure 7

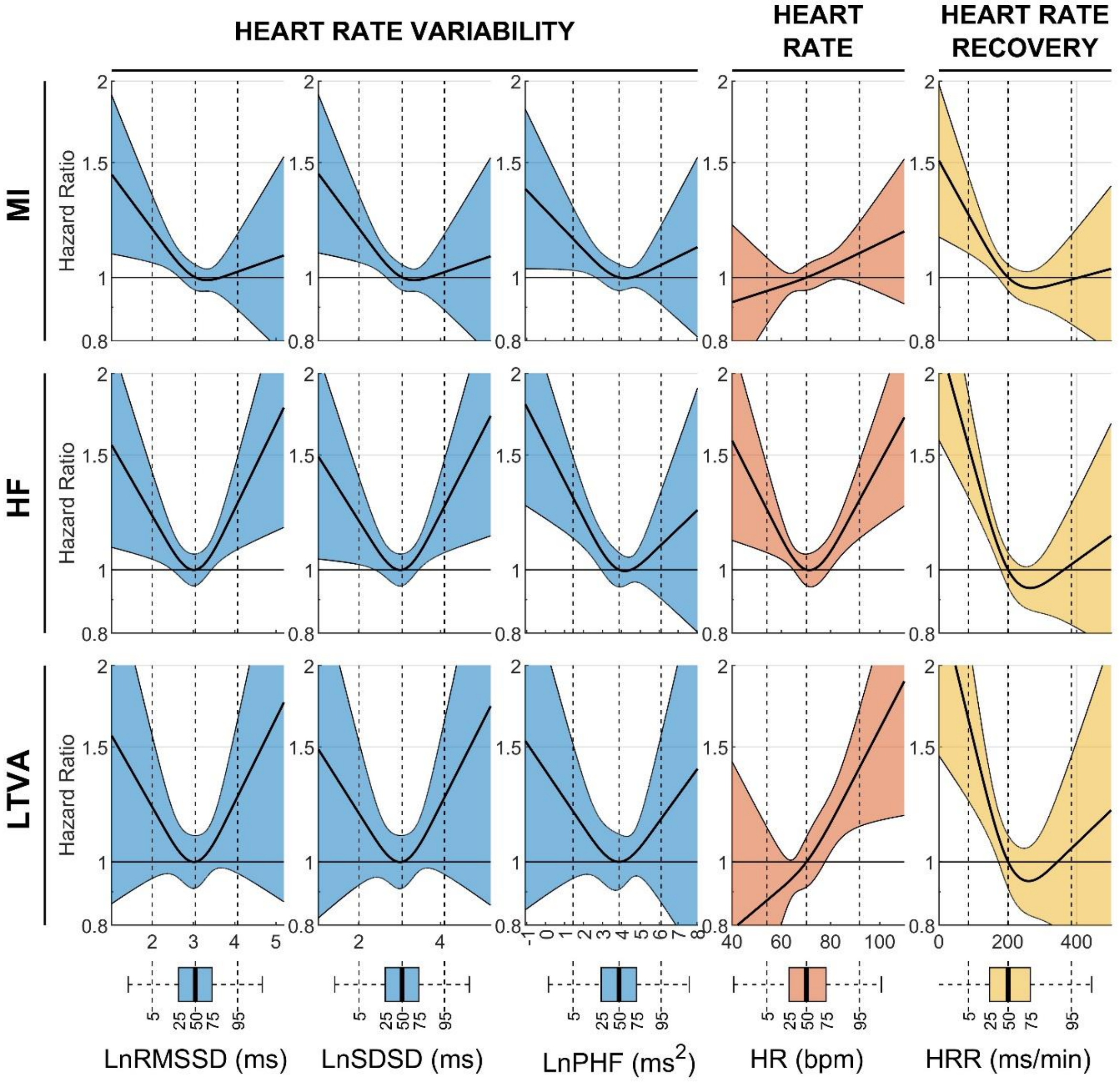

Supplementary Figure 7: Hazard ratio and 95% confidence interval (shaded area) for myocardial infarction (MI), heart failure (HF) and life-threatening ventricular arrhythmia (LTVA) as a function of all ultra-short HRV indices: Cox regression models use restricted cubic splines to model the exposures and are fully adjusted (see text).

Supplementary Figure 8

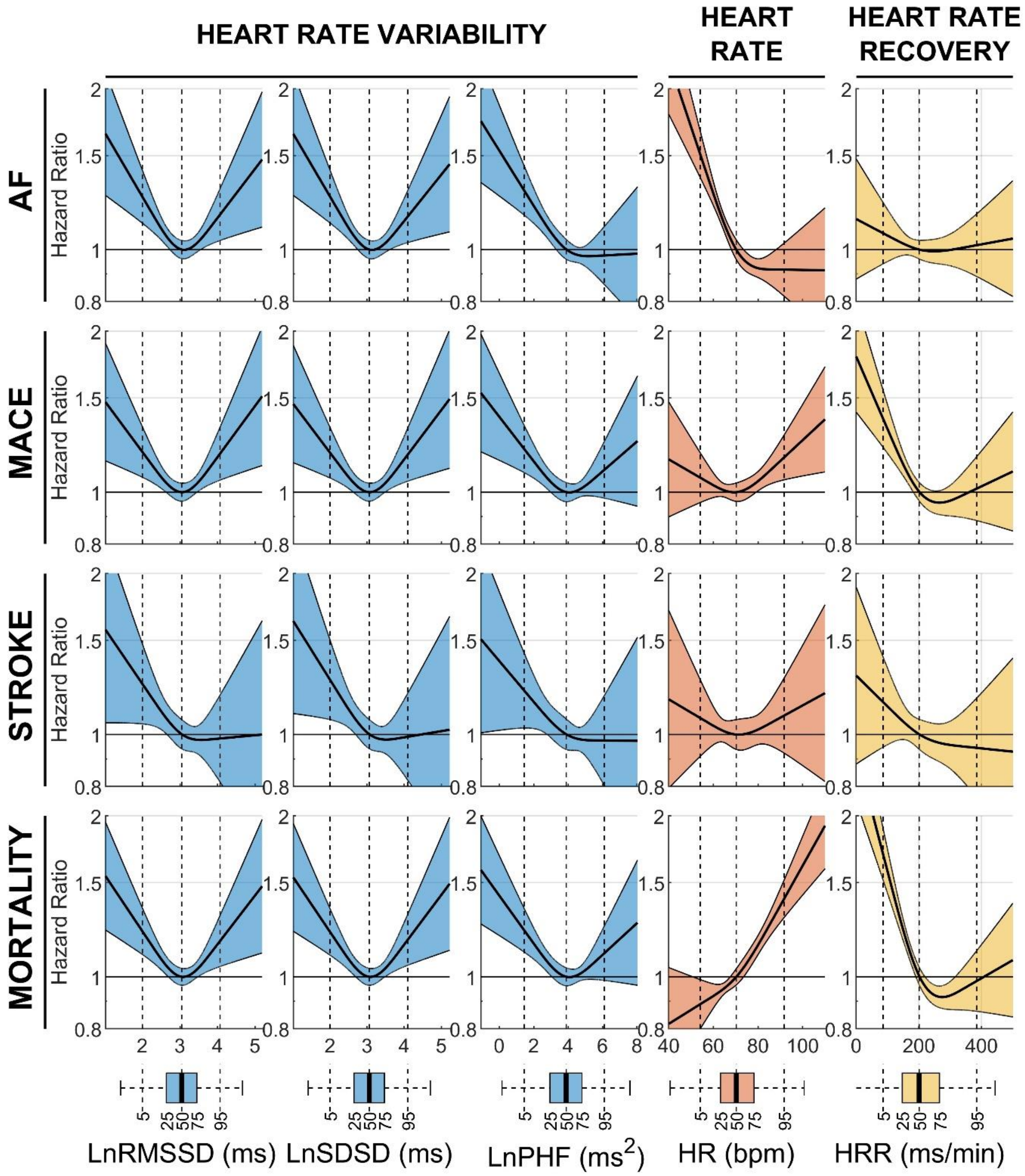

Supplementary Figure 8: Results of sensitivity analysis. Hazard ratio and 95% confidence interval (shaded area) for atrial fibrillation (AF), major adverse cardiovascular events (MACE), stroke and mortality as a function of resting heart rate (HR), ultra-short heart rate variability (usHRV) and heart rate recovery (HRR). Results obtained after excluding participants who were taking beta-blockers medication or who had diabetes at the time of testing.

Supplementary Figure 9

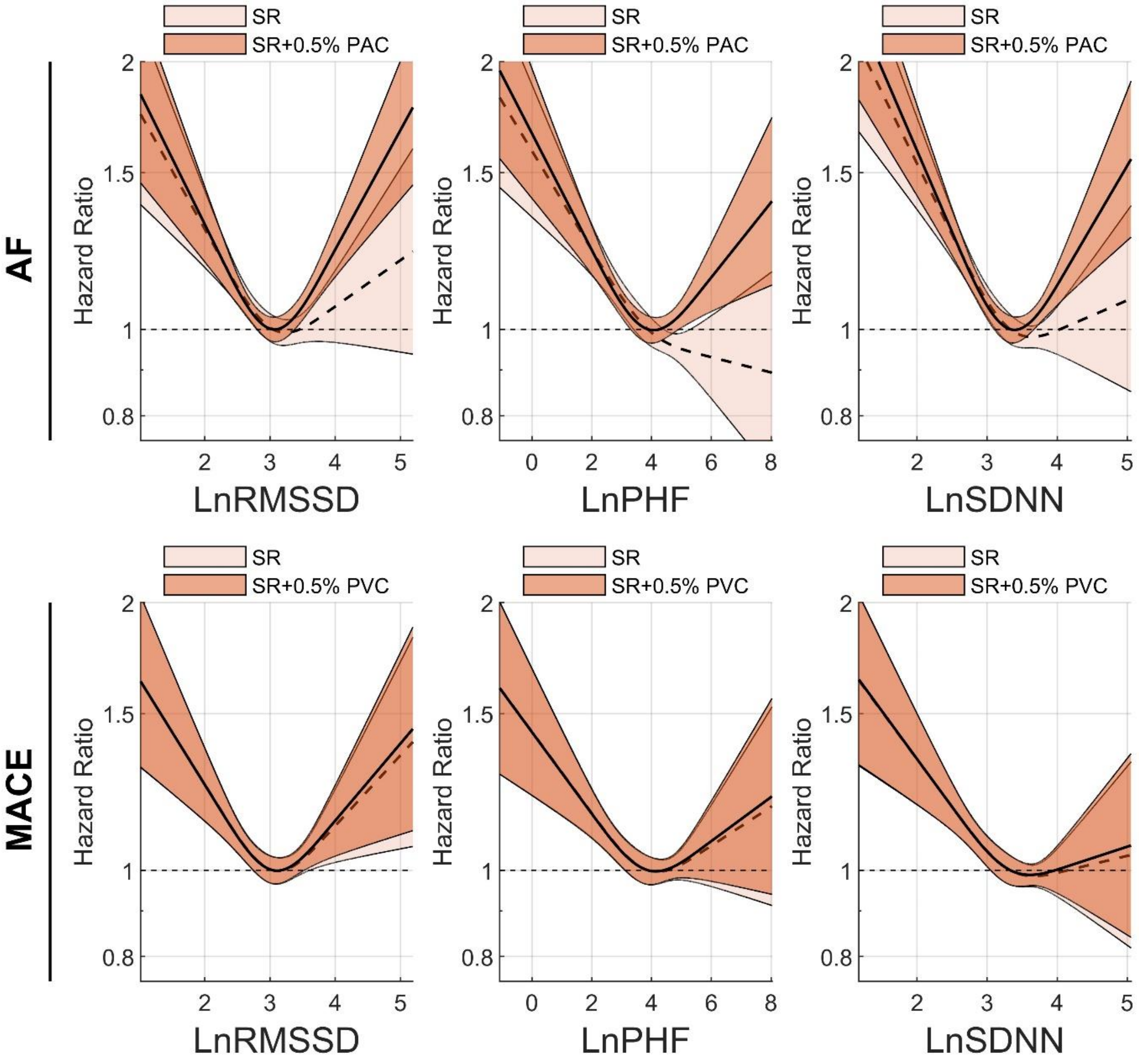

Supplementary Figure 9: Effect of adding individuals previously excluded for showing premature atrial (above) or ventricular (below) contractions to the study population. Dashed line and transparent areas represent hazard ratio and confidence intervals for individuals in sinus rhythm also shown in Figure 5. Solid line and dark areas represent hazard ratio and confidence intervals after including n=300 individuals (0.5%) without known cardiovascular disease but showing at least one premature contraction in the 15 sec resting ECG.
