# Supplementary material for: Long-term association of ultra-short heart rate variability with cardiovascular events
Source: Sci Rep. 2023 Nov 3;13:18966. doi: 10.1038/s41598-023-45988-2 (PMC10624663; doi:10.1038/s41598-023-45988-2)
Supplement: Supplementary file 2 — Supplementary Information 2. [file 41598_2023_45988_MOESM2_ESM.pdf]

Supplementary Table 1: Codes for diagnoses of cardiovascular events used for excluding participant with prevalent cardiovascular disease at baseline

Subgroup	ICD9 codes	ICD10 codes	Definition
Ischemic Heart Disease	411.1	I20	Unstable angina
		I21	Acute myocardial infarction
	410.11	I21.0	Acute transmural myocardial infarction of anterior wall
	410.41	I21.1	Acute transmural myocardial infarction of inferior wall
	410.81	I21.2	Acute transmural myocardial infarction of other sites
	410.91	I21.3	Acute transmural myocardial infarction of unspecified site
	410.71	I21.4	Acute subendocardial myocardial infarction
	410.91	I21.9	Acute myocardial infarction, unspecified
		I22	Subsequent myocardial infarction
	410.01/410.11	I22.0	Subsequent myocardial infarction of anterior wall
	410.21/410.31/410.41	I22.1	Subsequent myocardial infarction of inferior wall
	410.51/410.61/410.81	I22.8	Subsequent myocardial infarction of other sites
	410.91	I22.9	Subsequent myocardial infarction of unspecified site
		I24	Other acute ischaemic heart diseases
		I24.0	Coronary thrombosis not resulting in myocardial infarction
	411.89	I24.8	Other forms of acute ischaemic heart disease
	411.89	I24.9	Acute ischaemic heart disease, unspecified
		I25	Chronic ischaemic heart disease
	429.2	I25.0	Atherosclerotic cardiovascular disease, so described
	414.0	I25.1	Atherosclerotic heart disease
		I25.2	Old myocardial infarction
	414.10/414.19	I25.3	Aneurysm of heart
		I25.4	Coronary artery aneurysm
	414.8	I25.5	Ischaemic cardiomyopathy
	414.8	I25.6	Silent myocardial ischaemia
		I25.8	Other forms of chronic ischaemic heart disease
	414.8/414.9	I25.9	Chronic ischaemic heart disease, unspecified
Cardiomyopathies		I42	Cardiomyopathy
	425.4	I42.0	Dilated cardiomyopathy
	425.11	I42.1	Obstructive hypertrophic cardiomyopathy
	425.18	I42.2	Other hypertrophic cardiomyopathy
	425	I42.3	Endomyocardial (eosinophilic) disease
	425.3	I42.4	Endocardial fibroelastosis
	425.4	I42.5	Other restrictive cardiomyopathy
	425.5	I42.6	Alcoholic cardiomyopathy
	425.9	I42.7	Cardiomyopathy due to drugs and other external agents
	425.2/425.4	I42.8	Other cardiomyopathies
	425.4/425.9	I42.9	Cardiomyopathy, unspecified
	425.8	I43	Cardiomyopathy in diseases classified elsewhere
		I43.0	Cardiomyopathy in infectious and parasitic diseases classified elsewhere
	425.7	I43.1	Cardiomyopathy in metabolic diseases
	425.7	I43.2	Cardiomyopathy in nutritional diseases
	425.8	I43.8	Cardiomyopathy in other diseases classified elsewhere
Heart Failure		I50	Heart failure
	428.0	I50.0	Congestive heart failure
	428.1	I50.1	Left ventricular failure
	428.0/428.9	I50.9	Heart failure, unspecified
Atrial Arrhythmia	427.3	I48	Atrial fibrillation
	427.31	I48.0	Paroxysmal atrial fibrillation
	427.31	I48.1	Persistent atrial fibrillation
	427.31	I48.2	Chronic atrial fibrillation
	427.32	I48.3	Typical atrial flutter
	427.32	I48.4	Atypical atrial flutter
		I48.9	Atrial fibrillation and atrial flutter, unspecified
Ventricular Arrhythmia	427.1	I47.2	Ventricular tachycardia
		I49.0	Ventricular fibrillation and flutter
Significant conduction disease		I44.1	Atrioventricular block, second degree
		I44.2	Atrioventricular block, complete
Arrhythmia general	427.9	I49.9	Cardiac arrhythmia, unspecified
Cardiac Arrest	427.5	I46.1	Sudden cardiac death, so described
	427.5	I46.9	Cardiac arrest, unspecified
SCD	427.5	I46.1	Sudden cardiac death, so described
Ischaemic Stroke		I63	Cerebral infarction
	434.91	I63.0	Cerebral infarction due to thrombosis of precerebral arteries
	434.91	I63.1	Cerebral infarction due to embolism of precerebral arteries
	434.91	I63.2	Cerebral infarction due to unspecified occlusion or stenosis of precerebral arteries
	434.01	I63.3	Cerebral infarction due to thrombosis of cerebral arteries
	434.11	I63.4	Cerebral infarction due to embolism of cerebral arteries
	434.91	I63.5	Cerebral infarction due to unspecified occlusion or stenosis of cerebral arteries
		I63.8	Other cerebral infarction
	434.91	I63.9	Cerebral infarction, unspecified
Haemorrhagic stroke		I61	Intracerebral haemorrhage
		I61.0	Intracerebral haemorrhage in hemisphere, subcortical
		I61.1	Intracerebral haemorrhage in hemisphere, cortical
		I61.2	Intracerebral haemorrhage in hemisphere, unspecified
		I61.3	Intracerebral haemorrhage in brain stem
		I61.4	Intracerebral haemorrhage in cerebellum
		I61.5	Intracerebral haemorrhage, intraventricular
		I61.6	Intracerebral haemorrhage, multiple localised
		I61.8	Other intracerebral haemorrhage
		I61.9	Intracerebral haemorrhage, unspecified
Unspecified stroke	436	I64	Stroke, not specified as haemorrhage or infarction
Aortic/Peripheral vascular disease		I70	Atherosclerosis
		I70.0	Atherosclerosis of aorta

		I70.00	Atherosclerosis of aorta (without gangrene)
		I70.01	Atherosclerosis of aorta (with gangrene)
		I70.2	Atherosclerosis of arteries of the extremities
		I70.20	Atherosclerosis of arteries of extremities (without gangrene)
		I70.21	Atherosclerosis of arteries of extremities (with gangrene)
		I70.8	Atherosclerosis of other arteries
		I70.80	Atherosclerosis of other arteries (without gangrene)
		I73	Other peripheral vascular diseases
		I73.0	Raynaud's syndrome
		I73.1	Thromboangiitis obliterans [Buerger]
		I73.8	Other specified peripheral vascular diseases
		I73.9	Peripheral vascular disease, unspecified
		I74	Arterial embolism and thrombosis
		I74.0	Embolism and thrombosis of abdominal aorta
		I74.1	Embolism and thrombosis of other and unspecified parts of aorta
		I74.2	Embolism and thrombosis of arteries of the upper extremities
		I74.3	Embolism and thrombosis of arteries of the lower extremities
		I74.4	Embolism and thrombosis of arteries of extremities, unspecified
		I74.5	Embolism and thrombosis of iliac artery
		I74.8	Embolism and thrombosis of other arteries
		I74.9	Embolism and thrombosis of unspecified artery
Other vascular disease		I71	Aortic aneurysm and dissection
		I71.0	Dissection of aorta [any part]
		I71.1	Thoracic aortic aneurysm, ruptured
		I71.2	Thoracic aortic aneurysm, without mention of rupture
		I71.3	Abdominal aortic aneurysm, ruptured
		I71.4	Abdominal aortic aneurysm, without mention of rupture
		I71.5	Thoracoabdominal aortic aneurysm, ruptured
		I71.6	Thoracoabdominal aortic aneurysm, without mention of rupture
		I71.8	Aortic aneurysm of unspecified site, ruptured
		I71.9	Aortic aneurysm of unspecified site, without mention of rupture
Hypertensive heart disease		I11	Hypertensive heart disease
		I11.0	Hypertensive heart disease with (congestive) heart failure
		I11.9	Hypertensive heart disease without (congestive) heart failure
Non-rheumatic valvular heart disease		I34	Nonrheumatic mitral valve disorders
		I34.0	Mitral (valve) insufficiency
		I34.1	Mitral (valve) prolapse
		I34.2	Nonrheumatic mitral (valve) stenosis
		I34.8	Other nonrheumatic mitral valve disorders
		I34.9	Nonrheumatic mitral valve disorder, unspecified
		I35	Nonrheumatic aortic valve disorders
		I35.0	Aortic (valve) stenosis
		I35.1	Aortic (valve) insufficiency
		I35.2	Aortic (valve) stenosis with insufficiency
		I35.8	Other aortic valve disorders
		I35.9	Aortic valve disorder, unspecified
		I36	Nonrheumatic tricuspid valve disorders
		I36.0	Nonrheumatic tricuspid (valve) stenosis
		I36.1	Nonrheumatic tricuspid (valve) insufficiency
		I36.8	Other nonrheumatic tricuspid valve disorders
		I36.9	Nonrheumatic tricuspid valve disorder, unspecified
		I37	Pulmonary valve disorders
		I37.0	Pulmonary valve stenosis
		I37.1	Pulmonary valve insufficiency
		I37.2	Pulmonary valve stenosis with insufficiency
		I37.8	Other pulmonary valve disorders
		I37.9	Pulmonary valve disorder, unspecified
Congenital heart disease		Q20	Congenital malformations of cardiac chambers and connexions
		Q20.0	Common arterial trunk
		Q20.1	Double outlet right ventricle
		Q20.2	Double outlet left ventricle
		Q20.3	Discordant ventriculoarterial connexion
		Q20.4	Double inlet ventricle
		Q20.5	Discordant atrioventricular connexion
		Q20.6	Isomerism of atrial appendages
		Q20.8	Other congenital malformations of cardiac chambers and connexions
		Q20.9	Congenital malformation of cardiac chambers and connexions, unspecified
		Q21	Congenital malformations of cardiac septa
		Q21.0	Ventricular septal defect
		Q21.1	Atrial septal defect
		Q21.2	Atrioventricular septal defect
		Q21.3	Tetralogy of Fallot
		Q21.4	Aortopulmonary septal defect
		Q21.8	Other congenital malformations of cardiac septa
		Q21.9	Congenital malformation of cardiac septum, unspecified
		Q22	Congenital malformations of pulmonary and tricuspid valves
		Q22.1	Congenital pulmonary valve stenosis
		Q22.2	Congenital pulmonary valve insufficiency
		Q22.4	Congenital tricuspid stenosis
		Q22.5	Ebstein's anomaly
		Q22.8	Other congenital malformations of tricuspid valve
		Q22.9	Congenital malformation of tricuspid valve, unspecified
		Q23	Congenital malformations of aortic and mitral valves
		Q23.0	Congenital stenosis of aortic valve
		Q23.1	Congenital insufficiency of aortic valve
		Q23.2	Congenital mitral stenosis
		Q23.3	Congenital mitral insufficiency

		Q23.4	Hypoplastic left heart syndrome
		Q23.8	Other congenital malformations of aortic and mitral valves
		Q23.9	Congenital malformation of aortic and mitral valves, unspecified
		Q24	Other congenital malformations of heart
		Q24.0	Dextrocardia
		Q24.1	Levocardia
		Q24.3	Pulmonary infundibular stenosis
		Q24.4	Congenital subaortic stenosis
		Q24.5	Malformation of coronary vessels
		Q24.6	Congenital heart block
		Q24.8	Other specified congenital malformations of heart
		Q24.9	Congenital malformation of the heart, unspecified
Myocarditis		B33.2	Viral carditis
		I40.0	Acute myocarditis
		I40.1	Isolated myocarditis
		I40.8	Other acute myocarditis
		I40.9	Acute myocarditis, unspecified
		I41.1	Myocarditis in viral diseases classified elsewhere
		I41.2	Myocarditis in other infectious and parasitic diseases classified elsewhere
		I41.8	Myocarditis in other diseases classified elsewhere
		I51.4	Myocarditis, unspecified

Supplementary Table 2: Codes for Primary and Secondary Outcomes

**Atrial Fibrillation**	
ICD10 codes	
I48	Atrial fibrillation
I48.0	Paroxysmal atrial fibrillation
I48.1	Persistent atrial fibrillation
I48.2	Chronic atrial fibrillation
I48.3	Typical atrial flutter
I48.4	Atypical atrial flutter
I48.9	Atrial fibrillation and atrial flutter, unspecified
Operative procedures	
K62.1	Percutaneous transluminal ablation of pulmonary vein to left atrium conducting system
K62.2	Percutaneous transluminal ablation of atrial wall for atrial flutter
K62.3	Percutaneous transluminal ablation of conducting system of heart for atrial flutter NEC
K62.4	Percutaneous transluminal internal cardioversion NEC
ICD9 codes	
4273	Atrial fibrillation and flutter

**Myocardial Infarction**	
ICD10 codes	
I21	Acute myocardial infarction
I21.0	Acute transmural myocardial infarction of anterior wall
I21.1	Acute transmural myocardial infarction of inferior wall
I21.2	Acute transmural myocardial infarction of other sites
I21.3	Acute transmural myocardial infarction of unspecified site
I21.4	Acute subendocardial myocardial infarction
I21.9	Acute myocardial infarction, unspecified
I22	Subsequent myocardial infarction
I22.0	Subsequent myocardial infarction of anterior wall
I22.1	Subsequent myocardial infarction of inferior wall
I22.8	Subsequent myocardial infarction of other sites
I22.9	Subsequent myocardial infarction of unspecified site
I23	Certain current complications following acute myocardial infarction
I23.0	Haemopericardium as current complication following acute myocardial infarction
I23.1	Atrial septal defect as current complication following acute myocardial infarction
I23.2	Ventricular septal defect as current complication following acute myocardial infarction
I23.3	Rupture of cardiac wall without haemopericardium as current complication following acute myocardial infarction
I23.4	Rupture of chordae tendineae as current complication following acute myocardial infarction
I23.5	Rupture of papillary muscle as current complication following acute myocardial infarction
I23.6	Thrombosis of atrium , auricular appendage and ventricle as current complications following acute myocardial infarction
I23.8	Other current complications following acute myocardial infarction
ICD9 codes	
4109	Acute myocardial infarction
Operative procedures	
1070	Coronary angioplasty (ptca) + stent
1095	Coronary artery bypass grafts (cabg)
1523	Triple Heart bypass
OPCS4	
K40	Saphenous vein graft replacement of coronary artery
K40.1	Saphenous vein graft replacement of one coronary artery
K40.2	Saphenous vein graft replacement of two coronary arteries
K40.3	Saphenous vein graft replacement of three coronary arteries
K40.4	Saphenous vein graft replacement of four or more coronary arteries
K40.9	Unspecified saphenous vein graft replacement of coronary artery
K41	Other autograft replacement of coronary artery
K41.1	Autograft replacement of one coronary artery NEC
K41.2	Autograft replacement of two coronary arteries NEC
K41.3	Autograft replacement of three coronary arteries NEC
K41.4	Autograft replacement of four or more coronary arteries NEC
K42	Allograft replacement of coronary artery
K42.4	Allograft replacement of four or more coronary arteries
K44	Other replacement of coronary artery

Supplementary Table S3: Risk of usHRV per usHRV quintile using individuals in the third quintile (Q3) as reference. Models were adjusted for including age, sex, body mass index, hypertension, smoking, LDL cholesterol, diabetes, and use of beta-blockers. HR: Hazard ratio; CIl and CIu: Lower and uppur bounds of 95% confidence intervals. P: p-value.

	**Atrial Fibirillation**				**Major Adverce CV Events**				**Stroke**				**Mortality**			
	HR	CIl	CIu	P	HR	CIl	CIu	P	HR	CIl	CIu	P	HR	CIl	CIu	P
RMSSD Q1 vs Q3	1.29	1.10	1.50	**0.001**	1.29	1.10	1.51	**0.001**	1.20	0.95	1.50	0.126	1.16	1.01	1.34	**0.040**
RMSSD Q2 vs Q3	1.17	1.02	1.35	**0.026**	1.24	1.08	1.44	**0.003**	1.04	0.84	1.29	0.696	1.11	0.97	1.27	0.133
RMSSD Q4 vs Q3	0.92	0.78	1.07	0.268	1.16	0.99	1.36	0.058	0.98	0.78	1.23	0.873	1.01	0.87	1.17	0.917
RMSSD Q5 vs Q3	1.04	0.88	1.22	0.675	1.17	0.99	1.38	0.064	0.83	0.64	1.07	0.157	1.07	0.91	1.25	0.412
SDSD Q1 vs Q3	1.25	1.07	1.46	**0.004**	1.26	1.08	1.47	**0.004**	1.24	0.99	1.56	0.065	1.15	0.99	1.32	0.059
SDSD Q2 vs Q3	1.17	1.02	1.35	**0.027**	1.27	1.10	1.46	**0.001**	1.03	0.83	1.28	0.771	1.04	0.91	1.19	0.573
SDSD Q4 vs Q3	0.89	0.76	1.04	0.159	1.17	1.00	1.36	0.052	0.97	0.77	1.22	0.795	0.98	0.84	1.14	0.793
SDSD Q5 vs Q3	1.00	0.85	1.18	0.958	1.19	1.00	1.40	**0.044**	0.84	0.65	1.09	0.188	1.04	0.88	1.21	0.659
PHF Q1 vs Q3	1.26	1.09	1.45	**0.002**	1.29	1.12	1.49	**0.001**	1.10	0.88	1.36	0.410	1.17	1.02	1.34	**0.025**
PHF Q2 vs Q3	1.05	0.92	1.21	0.466	1.07	0.92	1.23	0.381	1.04	0.84	1.29	0.690	1.06	0.92	1.21	0.423
PHF Q4 vs Q3	0.90	0.77	1.05	0.168	1.10	0.94	1.27	0.236	0.97	0.77	1.22	0.795	1.03	0.89	1.19	0.664
PHF Q5 vs Q3	0.83	0.70	0.98	**0.030**	1.08	0.92	1.28	0.333	0.96	0.74	1.23	0.730	1.02	0.87	1.20	0.792
HR_bpm Q1 vs Q3	1.38	1.20	1.58	**0.000**	1.11	0.96	1.28	0.153	0.93	0.75	1.14	0.470	0.90	0.78	1.04	0.150
HR_bpm Q2 vs Q3	1.21	1.05	1.39	**0.009**	0.94	0.81	1.08	0.385	0.86	0.70	1.06	0.163	0.93	0.82	1.07	0.332
HR_bpm Q4 vs Q3	0.92	0.79	1.07	0.293	1.05	0.91	1.21	0.478	0.84	0.68	1.04	0.105	1.03	0.90	1.18	0.664
HR_bpm Q5 vs Q3	0.95	0.81	1.10	0.472	1.16	1.01	1.33	**0.033**	0.92	0.74	1.13	0.425	1.30	1.14	1.47	**0.000**
DRRrec Q1 vs Q3	1.04	0.87	1.23	0.680	1.35	1.14	1.58	**0.000**	1.02	0.79	1.31	0.878	1.37	1.17	1.59	**0.000**
DRRrec Q2 vs Q3	1.01	0.87	1.17	0.934	1.04	0.89	1.20	0.644	1.02	0.82	1.26	0.891	1.06	0.92	1.23	0.410
DRRrec Q4 vs Q3	1.04	0.90	1.21	0.588	0.96	0.82	1.12	0.600	0.89	0.71	1.13	0.345	0.88	0.75	1.02	0.096
DRRrec Q5 vs Q3	1.02	0.85	1.23	0.797	0.94	0.78	1.14	0.543	0.88	0.66	1.16	0.363	0.99	0.83	1.20	0.950

Supplementary Table 4: NSHD Data Distribution

Parameter	Data
Age (years)	63.7 (63.0, 64.3)
Sex (male)	631 (45.9%)
BMI (Kg/m2)	26.9 (24.4, 30.1)
Resting HR (bpm)	63.8 (57.8, 70.7)
Hypertension (yes)	554 (60.3%)
T2 Diabetes	180 (13.1%)
RMSSD (ms)	20.6 (14.3, 29.7)
SDSD (ms)	20.6 (14.3, 29.7)
PHF (ms2)	59.1 (30.6, 120.0)

Continuous data shown as median (interquartile range), binary variables shown as number (%)
